# Validity of Fecal Occult Blood Test in the National Cancer Screening Program, Korea

**DOI:** 10.1371/journal.pone.0079292

**Published:** 2013-11-08

**Authors:** Aesun Shin, Kui Son Choi, Jae Kwan Jun, Dai Keun Noh, Mina Suh, Kyu-Won Jung, Byung Chang Kim, Jae Hwan Oh, Eun-Cheol Park

**Affiliations:** 1 Molecular Epidemiology Branch, Research Institute, National Cancer Center, Goyang-si, Korea; 2 Cancer Registration & Statistics Branch, National Cancer Control Institute, National Cancer Center, Goyang-si, Korea; 3 Cancer Early Detection Branch, National Cancer Control Institute, National Cancer Center, Goyang-si, Korea; 4 Center for Colorectal Cancer, Hospital, National Cancer Center, Goyang-si, Korea; 5 Department of Preventive Medicine & Institute of Health Services Research, Yonsei University, Seoul, Korea; University General Hospital of Heraklion and Laboratory of Tumor Cell Biology, School of Medicine, University of Crete, Greece

## Abstract

**Aim:**

The aims of the current study were to assess the validity of the fecal occult blood test (FOBT) in an organized screening setting in Korea and to determine factors associated with FOBT validity, such as screening round, age group, and anatomical location of the cancer.

**Methods:**

Study participants were those who were 50 years and older who received an FOBT through the National Cancer Screening Program between 2004 and 2007. Colorectal cancer diagnoses were ascertained through linkage with the Korean National Cancer Incidence Database. The positivity rate, colorectal cancer detection rate, interval cancer rate, sensitivity, specificity, and positive predictive value of the FOBT were calculated.

**Results:**

A total of 2,193,093 tests were included in the analysis. Overall, the sensitivity of the FOBT for colorectal cancer was 59.7% for the first round and 56.1% for the subsequent round. Sensitivity was highest for distal colon cancer (65.9%) in the first round, and for rectal cancer (58.4%) for the subsequent round. The sensitivity and positive predictive value of the FOBT generally improved between 2004 and 2008.

**Conclusions:**

The FOBT showed reasonable validity in an organized screening setting, and the validity of the FOBT varied by screening round, anatomical location, and screening year.

## Introduction

Colorectal cancer (CRC) incidence and mortality has been increasing in Korea. The age-standardized mortality rate for CRC was 1.7 per 100,000 in 1983 and increased to 17.4 in 2010 in men and increased from 1.6 in 1983 to 13.5 in 2010 in women [1]. A joint regression analysis showed that mortality rates increased 7.7% annually between 1983 and 2002, then stabilized in men. Similarly, mortality rates increased 9.1% annually between 1983 and 1994, and 4.2% between 1994 and 2004 then stabilized in women [2]. Annual percent changes in age-standardized incidence rates for CRC were 7% in men and 5.3% in women between 1999 and 2008 [3]. As a result, by 2009, CRC had become the second most common cancer in men and the third most common cancer in women [4].

The rapid increase in CRC incidence could be explained not only by changes in risk factors, but also by the dissemination of CRC screening. Based on evidence that screening reduces CRC mortality [5]–[8], national guidelines in several countries now recommend regular CRC screening for average-risk persons aged 50 years or older using one or more of the following options: an annual fecal occult blood test (FOBT), flexible sigmoidoscopy every 5 years, a combination of FOBT and flexible sigmoidoscopy, colonoscopy every 10 years, and/or double-contrast barium enema (DCBE) every 5 years [9]. Recently, the Asia Pacific Working Group on CRC reached a consensus to develop guidelines for CRC screening and recommended the FOBT, flexible sigmoidoscopy, and colonoscopy as the best options [10]. In Korea, the government introduced nationwide CRC screening as part of the National Cancer Screening Program (NCSP) for Medical Aid recipients and National Health Insurance (NHI) beneficiaries in the lower 50% income bracket in 2004. The NCSP provides an annual FOBT free of charge as the primary screening method for men and women aged 50 years or older. In FOBT-positive individuals, the NCSP also provides follow-up investigation by either colonoscopy or DCBE [11].

In population-based, organized screening settings with cancer case ascertainment by linkage to a cancer registry database, test sensitivity for the latex agglutination FOBT was 73.8% in an Italian study [12]. Several factors have been associated with the validity of the FOBT. The average FOBT hemoglobin content among adenoma patients was higher for older age groups and those with a left-side colon tumor after adjustment for the clinical characteristics of adenoma [13]. In a systematic review of prospective studies that were conducted among average-risk adults, all of whom had an FOBT and colonoscopy, and that reported site-specific FOBT sensitivity for advanced neoplasia, 5 out of 8 studies showed that the FOBT had higher sensitivity for left-sided advanced neoplasia, although the confidence intervals overlapped due to limited sample sizes [14].

The objective of the current study was to assess the validity of the FOBT in the NCSP setting in Korea and to determine factors associated with FOBT validity, such as screening round, screening year, sex, age group, FOBT kit (qualitative/quantitative), and anatomical location of the cancer.

## Materials and Methods

### Study population

Data were obtained from the NCSP database, which contains information on Medical Aid recipients and on the NHI beneficiaries invited to participate in the NCSP. The NCSP provides an annual CRC screening through a fecal immunochemical test (FIT; qualitative or quantitative) for men and women aged 50 years and older. Within the framework of the NCSP, eligible men and women receive an invitation letter from the NHI Corporation (NHIC) at the beginning of each calendar year. In 2004, Medical Aid recipients and NHI beneficiaries in the lower 30% income bracket were eligible for the program. In 2005, the NCSP expanded its target population to the lower 50% income bracket. All of the target population receives an invitation letter from the NHIC at the beginning of the year.

In the colorectal cancer screening Program, FOBT was performed as a primary screening test by using one-day fecal immunochemical test instead of guaiac FOBT. FIT tests use specific antibodies against human blood components. For immunochemical FOBT, stool samples were collected by the individuals themselves either at home or at a screening center. In the laboratory, immunochemical FOBT is performed either qualitative (positive/negative) or quantitative (measurement of fecal hemoglobin content) method. Qualitative immunochemical FOBTs use immunochromatographic technology, and it allows simple and office-based analysis. Quantitative immunochemical FOBTs have important advantages over qualitative FOBTs in terms of their automation, which removes interobserver variation in the interpretation of test results, improving reproducibility and allowing for high-throughput testing. Four commercially available qualitative immunochemical FOBT kits (OC-Hemocatch Lignt TM, Eiken Chemical Co., Japan, cut-off point of 50 ng/ml; FOB test, Humasis Co., Korea, cut-off point of 50 ng/ml; SD Bioline FOB, SD Co., Korea, cut-off point of 30 ng/ml; ASAN Easy Test FOB, Asan Pharm Co., Korea, cut-off point of 50 ng/ml) are widely used in Korea. Regarding quantitative immunochemical FOBT, OC sensor method by latex agglutination nephelometric immunoassay (Eiken Chemical Co. Japan) is most popularly used. Each colorectal cancer screening unit chose which method to be used in their unit. In 2009, 72.8% of participants received a qualitative FIT and 27.2% received a quantitative FIT through NCSP for colorectal cancer.

Participants were notified of the FOBT results (reported as ‘positive’ or ‘negative’). Participants who had a positive FOBT result were contacted by telephone by the medical staff, informed of the positive test result, and offered an appointment date for follow-up testing by either colonoscopy or DCBE free of charge. FOBT-positive participants could choose their preferred follow-up test. All of these examinations were performed free of charge at a clinic or hospital designated as a CRC screening unit by the NHIC. To be designated as a CRC screening unit, a clinic or hospital must have colonoscopy equipment and at least one full-time medical doctor. CRC screening units must be able to conduct not only FOBT testing but also follow-up colonoscopy.

The population for the current study was restricted to men and women aged 50 years or older who were invited to participate in the NCSP for CRC screening between January 1, 2004 and December 31, 2007. Excluded from the analysis were 528 participants with missing screening results. The first participation to the NCSP was defined as the first screening, regardless of screening experience outside the NCSP. Any screening after the first screening was defined as consequent screening. The final study sample consisted of 1,809,139 participants and 2,193,093 FOBTs. The current study used the NCSP databases, which include informed consent for the collection of their screening results and health data, obtaining informed consent for this specific study was waived as the NCSP database was so large; this study was approved by the institutional review board of the National Cancer Center, Korea.

### Ascertainment of outcome

The final CRC diagnosis, tumor stage, and histopathological information were ascertained through linkage with the Korea National Cancer Incidence Database (KNCIDB) at the Korea Central Cancer Registry (KCCR). The KNCIDB contains 95% of newly diagnosed malignancies in Korea [15]. We used the CRC diagnoses reported to the KNCIDB through December 2008, allowing 12 months after the initial screening for the diagnostic work-up to be completed and the results to be fully reported.

Anatomical subsites were defined based on the tenth version of the International Statistical Classification of Diseases and Related Health Problems (ICD-10) [16]. The proximal colon was defined as the cecum (C18.0), appendix (C18.1), ascending colon (C18.2), hepatic flexure (C18.3), transverse colon (C18.4), and splenic flexure of the colon (C18.5); the distal colon was defined as the descending colon (C18.6) and sigmoid colon (C18.7). The rectum included the rectosigmoid colon (C19) and rectum (C20). Overlapping lesions of the colon (C18.8) and colon not otherwise specified (C18.9) were not included in the subsite analysis.

### Statistical analysis

We calculated the following performance measures of the FOBT examinations: positivity rates, CRC detection rates, interval cancer rates, sensitivity, specificity, and positive predictive values (PPVs) for detecting CRC. CRC detection rates were calculated as the number of CRCs detected per 1,000 FOBT examinations within a year from the time of a positive screening in the NCSP. Screening-detected cancer was defined as a cancer registered to the Korea Central Cancer Registry within one year of positive FOBT. Interval cancer rates were calculated as the number of CRCs diagnosed within 1 year of a negative screening per 1,000 negative examination results. Interval cancer was defined as a CRC cancer that was diagnosed outside a screening program within a year from the time of a negative screening in the NCSP. PPV (*i.e.*, the probability of a cancer diagnosis within 1 year after a positive screening examination) was estimated as the number of cancers diagnosed per 100 positive examination results. Program sensitivity was defined as the probability of a positive FOBT screening, given a finding of cancer within 1 year after a screening [true positive / (true positive + false negative)]. Program specificity was defined as the probability of a negative FOBT screening, given no finding of cancer within 1 year after a screening [true negative / (true negative + false positive)].

Performance measures were stratified by first (prevalence) and subsequent (incidence) examinations. The first CRC screening that a participant underwent was designated as the first round, regardless of any previous CRC screening. Additional screenings received by these participants were considered to be subsequent rounds. The results from subsequent CRC screenings were excluded for all participants with a final diagnosis of CRC or other cancer that had been identified during the first round of screening. SAS software (ver. 9.1; SAS Institute Inc., Cary, NC, USA) was used for all statistical calculations.

## Results

A total of 2,193,093 FOBTs were included in the analysis, of which 1,809,139 were a first screening and 383,954 were subsequent screenings (Table 1). A total of 225,755 FOBTs were administered and the numbers increased every year with 777,999 FOBTs administered in 2007. Fifty-five percent of FOBTs were administered to women. Use of qualitative FOBT kits increased each year.

**Table 1:** Characteristics of the screening participants (by each screening for participants with multiple screenings) of the National Cancer Screening Program, 2004-2007.

	FOBT kits		
	Qualitative	Quantitative	NA
Screening round			
First screening round	1,449,081 (83.2)	354,014 (79.8)	6,044 (85.0)
Subsequent screening round	293,544 (16.8)	89,340 (20.2)	1,070 (15.0)
Age (N, %)			
50-59	848,957 (48.7)	216,961 (48.9)	3,239 (45.5)
60-69	648,127 (37.2)	168,510 (38.0)	2,695 (37.9)
70+	245,541 (14.1)	57,883 (13.1)	1,180 (16.6)
Sex (N, %)			
Men	783,623 (45.0)	193,329 (43.6)	3,087 (43.4)
Women	959,002 (55.0)	250,025 (56.4)	4,027 (56.6)
Insurance type (N, %)			
MAP	216,441 (12.4)	48,776 (11.0)	2,007 (28.2)
NHI	1,526,184 (87.6)	394,578 (89.0)	5,107 (71.8)
Screening year			
2004	193,636 (11.1)	29,525 (6.6)	2,594 (36.5)
2005	422,253 (24.2)	75,401 (17.0)	766 (10.8)
2006	568,091 (32.6)	121,367 (27.4)	1,461 (20.5)
2007	558,645 (32.1)	217,061 (49.0)	2,293 (32.2)

MAP, Medical Aid program; NHI, National Health Insurance

Overall, FOBT positivity was 7.28% (Table 2). For the first screening round, the screening-detected cancer rate was 1.38 per 1,000 and interval cancer rate was 1.0 per 1,000. These rates were 1.22 and 1.03, respectively, for the subsequent round. Both screening-detected and interval cancer rates were higher for older age groups and for men. Use of the qualitative FOBT kit showed higher rates for screening-detected cancer, whereas the quantitative FOBT kit showed higher rates for interval cancer, although the differences were not statistically significant. Screening detected cancer rates were higher for both the first and subsequent rounds between 2004 and 2007, whereas interval cancer rates were lower between 2005 and 2007.

**Table 2:** Characteristics of screening-detected and interval colorectal cancer, the National Cancer Screening Program, Korea, 2004-2007.

		First screening round				Subsequent screening round			
		Screening-detected		Interval cancer		Screening-detected		Interval cancer	
	FOBT positivity, % (95% CI)	Number	Rates Per 1,000 (95% CI)	Number	Rates Per 1,000 (95% CI)	Number	Rates Per 1,000 (95% CI)	Number	Rates Per 1,000 (95% CI)
Overall	7.28 (7.25–7.32)	2491	1.38 (1.32–1.43)	1680	1.00 (0.96–1.05)	470	1.22 (1.12–1.34)	367	1.03 (0.93–1.14)
Age									
50–59	6.89 (6.84–6.94)	849	0.93 (0.87–1.00)	525	0.62 (0.57–0.68)	122	0.77 (0.64–0.92)	88	0.59 (0.48–0.73)
60–69	7.48 (7.42–7.54)	1067	1.63 (1.54–1.74)	719	1.19 (1.11–1.28)	234	1.41 (1.24–1.60)	170	1.10 (0.94–1.28)
70+	8.13 (8.03–8.23)	575	2.34 (2.16–2.54)	436	1.93 (1.76–2.13)	114	1.93 (1.60–2.33)	109	2.00 (1.65–2.42)
Sex									
Men	8.51 (8.45–8.56)	1615	2.03 (1.94–2.14)	993	1.37 (1.29–1.46)	326	1.75 (1.57–1.96)	249	1.45 (1.28–1.65)
Women	6.29 (6.25–6.34)	876	0.86 (0.81–0.92)	687	0.72 (0.67–0.78)	144	0.73 (0.62–0.86)	118	0.63 (0.53–0.76)
FOBT kit									
Qualitative	8.43 (8.38–8.47)	2049	1.41 (1.35–1.48)	1268	0.96 (0.90–1.01)	374	1.27 (1.15–1.41)	270	1.00 (0.89–1.13)
Quantitative	2.73 (2.68–2.77)	434	1.23 (1.11–1.35)	405	1.18 (1.07–1.30)	95	1.06 (0.86–1.31)	97	1.11 (0.91–1.36)
NA	11.36 (10.63–12.12)	8	1.32 (0.62–2.72)	7	1.32 (0.58–2.84)	1	0.93 (0.05–6.05)	0	0.00 (0.00–4.84)
Year of screening									
2004	7.07 (6.97–7.18)	240	1.06 (0.93–1.21)	242	1.15 (1.01–1.31)	0	NA	0	NA
2005	6.88 (6.81–6.95)	613	1.31 (1.21–1.42)	436	1.00 (0.91–1.10)	31	1.00 (0.69–1.43)	38	1.31 (0.94–1.82)
2006	7.78 (7.72–7.85)	797	1.38 (1.29–1.48)	506	0.95 (0.87–1.04)	134	1.18 (0.99–1.40)	118	1.12 (0.93–1.34)
2007	7.16 (7.10–7.22)	841	1.56 (1.46–1.67)	496	0.99 (0.91–1.09)	305	1.28(1.14–1.43)	211	0.95 (0.82–1.08)

Overall, the sensitivity of the FOBT was 59.7% for the first screening round and 56.1% for the subsequent round (Table 3). Sensitivity was higher for younger age groups, men, use of the qualitative kit, and more recent years. Sensitivity was highest for distal colon cancer, followed by rectal cancer and proximal colon cancer in the first screening round. However, for the subsequent screening round, sensitivities for distal colon cancer and rectal cancer were similar (Table 4).

**Table 3:** Validity of FOBT for colorectal cancer.

	First screening round			Subsequent screening round		
	Sensitivity, % (95% CI)	Specificity, % (95% CI)	Positive predictive value, % (95% CI)	Sensitivity, % (95% CI)	Specificity, % (95% CI)	Positive predictive value, % (95% CI)
Overall	59.72 (58.21–61.21)	92.75 (92.71–92.78)	1.87 (1.80–1.94)	56.15 (52.71–59.54)	93.26 (93.18–93.34)	1.79 (1.63–1.96)
Age						
50–59	61.79 (59.15–64.36)	93.09 (93.03–93.14)	1.33 (1.25–1.43)	58.10 (51.10–64.79)	93.78 (93.66–93.90)	1.22 (1.02–1.46)
60–69	59.74 (57.42–62.02)	92.56 (92.49–92.62)	2.15 (2.03–2.29)	57.92 (52.93–62.76)	93.07 (92.94–93.19)	2.00 (1.75–2.27)
70+	56.87 (53.75–59.94)	91.99 (91.88–92.09)	2.85 (2.63–3.09)	51.12 (44.38–57.83)	92.39 (92.17–92.60)	2.48 (2.06–2.99)
Sex						
Men	61.92 (60.03–63.79)	91.52 (91.46–91.58)	2.35 (2.24–2.47)	56.70 (52.53–60.77)	92.26 (92.13–92.38)	2.22 (1.99–2.47)
Women	56.05 (53.54–58.52)	93.70 (93.65–93.75)	1.35 (1.27–1.45)	54.96 (48.72–61.06)	94.20 (94.09–94.30)	1.24 (1.05–1.46)
FOBT kit						
Qualitative	61.77 (60.09–63.43)	91.65 (91.60–91.70)	1.67 (1.60–1.74)	58.07 (54.15–61.90)	91.91 (91.81–92.01)	1.55 (1.40–1.72)
Quantitative	51.73 (48.29–55.15)	97.31 (97.26–97.36)	4.37 (3.98–4.79)	49.48 (42.23–56.75)	97.70 (97.59–97.79)	4.42 (3.61–5.40)
NA	53.33 (27.42–77.72)	88.12 (87.27–88.92)	1.10 (0.51–2.26)	100.00 (5.46–100.00)	92.24 (90.43–93.74)	1.19 (0.06–7.37)
Year of screening						
2004	49.79 (45.25–54.34)	93.02 (92.91–93.12)	1.50 (1.32–1.71)	NA	NA	NA
2005	58.44 (55.38–61.43)	93.24 (93.17–93.32)	1.91 (1.76–2.07)	44.93 (33.10–57.32)	93.10 (92.81–93.38)	1.43 (0.99–2.04)
2006	61.17 (58.45–63.81)	92.26 (92.19–92.32)	1.76 (1.64–1.88)	53.17 (46.82–59.44)	92.74 (92.59–92.89)	1.60 (1.34–1.89)
2007	62.90 (60.24–65.49)	92.72 (92.65–92.79)	2.10(1.97–2.25)	59.11 (54.72–63.36)	93.53 (93.43–93.62)	1.94 (1.73–2.17)

**Table 4:** Validity of FOBT for colorectal cancer by anatomical location.

	First screening round			Subsequent screening round		
	Screening-detected,n, (%)	Interval cancern, (%)	Sensitivity% (95% CI)	Screening-detected,n, (%)	Interval cancern, (%)	Sensitivity% (95% CI)
Location						
Proximal colon	450 (18.07)	354 (21.07)	55.97 (52.46–59.43)	74 (15.74)	76 (20.71)	49.33 (41.13–57.58)
Distal colon	816 (32.76)	423 (25.18)	65.86 (63.13–68.49)	131 (27.87)	96 (26.16)	57.71(50.99–64.17)
Rectum	1032 (41.43)	773 (46.01)	57.17 (54.85–59.47)	229 (48.72)	163 (44.41)	58.42 (53.35–63.32)
NA	193 (7.75)	130 (7.74)	59.75 (54.16–65.11)	36 (7.66)	32 (8.72)	56.15 (40.54–65.01)

## Discussion

In the current study, the sensitivity of the FOBT for CRC was higher for the first round compared with the subsequent round, screening conducted in more recent years, among men, older age groups, and for distal colon cancer. A decrease in FOBT sensitivity in subsequent screening rounds was observed in an organized screening in Israel. In that study, sensitivities for detecting a cancer, adenoma, or polyp were 85.3% in the first round and 69.2% in the second round [17]. However, the sensitivity was higher for the subsequent round (84%) than the first round (57.3%) in an Italian study [12].

Immunochemical FOBT generally shows better sensitivity and PPV compared with guaiac FOBT [18]–[23]. Therefore, NCSP requires immunochemical FOBT as the primary screening modality. The proportion of quantitative immunochemical FOBT use within the NCSP compared with the qualitative immunochemical FOBT increased gradually between 2004 and 2007. Qualitative FOBT showed better sensitivity, whereas quantitative FOBT showed better specificity and PPV. Although the NCSP did not require reporting the commercial name of the FOBT used or the cut-off point, we assumed that the qualitative FOBT set a lower fecal Hb threshold than the quantitative FOBT [23], [24]. In addition, positivity rates of the FOBT in our study were relatively high compared with other studies of average-risk populations, where positivity rates range between 2.0% and 8.1% [20]–[22], [25]. The positivity rates for the qualitative FOBT were three times higher than those for the quantitative FOBT, and the transition to widespread use of the quantitative FOBT would further lead to an overall lower positivity rate in the future [26].

The sensitivity of the FOBT was higher for men than women, especially for the first screening round in our study. This result is consistent with the results from a Finnish randomized screening program, which showed a sensitivity of 61.8% in men and 47.8% in women [27], and with results from a population-based study in Italy, where sensitivities were 78.4% for men and 66.6% for women, although the differences were not statistically significant [12]. High PPV and sensitivity among men and older age groups were observed in a Dutch study [25]. The higher PPV and sensitivity for CRC detection found in men compared with women may be explained by the higher incidence rates of CRC and colorectal adenoma in men. In the Korean population, the male-to-female colorectal incidence rate ratios (IRR) were 1.29∼1.57 between 1999 and 2009 [4]. High PPV and sensitivity among older age groups may also be explained by age-specific incidence patterns.

In our study, the sensitivity of the FOBT for detecting CRC was highest for distal colon cancer and rectal cancer compared with proximal colon cancer. Our result is partly consistent with the Israeli study, in which the FOBT showed the highest sensitivity for the left and sigmoid colon (87.9%) and similar sensitivity for the right and transverse colon (78.6%) and rectum (78.6%) [17]. In contrast, sensitivities of the FOBT were not different by cancer subsite in an Italian study (72.7% for colon vs. 75.8% for rectum) [12]. The average FOBT Hb content has been shown to be higher for those who possess a left-sided colon adenoma compared with those who have a right-sided colon adenoma [13] or cancer [23]. However, the latter study showed high FOBT Hb content among patients with an advanced adenoma in the proximal colon compared with patients with an advanced adenoma in the distal colon [23]. Reasons suggested for the high sensitivity of the FOBT for distal colon cancer compared with proximal colon cancer are as follows: 1) a higher proportion of pedunculated adenomas in the left colon, which are more likely to have advanced histology, 2) solid stool in the left-sided colon causes mechanistic irritation to neoplasia and, thus, bleeding, and 3) hemoglobin, the molecular target of the FOBT, which originates from right-sided neoplasia is subjected to more degradation compared with hemoglobin originating from left-sided neoplasia [14]. As a result of the relatively low sensitivity of the FOBT for proximal colon cancer, the proportion of proximal colon cancers increased among interval cancers.

In the current study, the sensitivity of the FOBT for colorectal cancer was 60% for the first round and 56% for the subsequent round. The sensitivity of the FOBT test in the current study was similar with the previous studies conducted in other countries. According to the recent study, sensitivities for qualitative FOBT tests were ranged from 30% to 73% [28]. The Spain study using the OC-Light kit that specifically detects human haemogliblin with sensitivity of 50 ng/ml buffer and popularly used in Korea, showed sensitivity of 61% [29]. The large Japanese study of nearly 22,000 asymptomatic, average-risk patients who were given a quantitative immunochemical FOBT, the sensitivity of FOBT for detecting invasive cancer was 65.8% [30]. The results of meta-analysis demonstrated the sensitivity of fecal immunochemical test was 67% (95% CI: 61%–73%) [31]. Generally, it is difficult to directly compare the results of different studies due to differences in the target populations and different methods used in the colorectal cancer screening programs. In addition, most previous studies included participants who underwent one-time FOBT and colonoscopy screening in clinical research settings, whereas our study was based on a population-based screening program. Therefore, comparison by screening intervals or screening rounds was not applicable. However, the sensitivity of FOBT test in the current study was within the rages reported by other studies.

Although sensitivity of FOBT in the current study was similar with others, the effort to increase accuracy of FOBT test is needed. Specifically, definition of cutoff is critical issue immunochemical FOBT tests and should be redefined for several of the tests to limit false-positive rates in population-based screening.

Limitations of the current study include the lack of complete information on colonoscopy after a positive FOBT. If FOBT-positive participants received a follow-up colonoscopy outside of the NCSP, the colonoscopy findings were not reported to the NCSP. Therefore, we could not assess the actual proportion of colonoscopy follow-up after an FOBT-positive screening. Low follow-up rates may have lead to an underestimation of screening-detected CRC rates. The follow-up rates among FOBT-positive participants were 39∼61% between 2004 and 2008 [32]. Lack of colonoscopy follow-up may also have lead to contamination of interval cancers detected during the subsequent round with missed cancer after positive FOBT due to lack of follow-up colonoscopy [33]. However, since the NSCP requires colonoscopy rather than sigmoidoscopy as a follow-up modality, systemic under-detection for proximal colon cancer should be minimized. In addition, due to limitations in detailed clinical information on follow-up colonoscopy findings, we could not use colorectal polyps as an outcome for the calculated test validity. Including colorectal polyps as an outcome would lead to better sensitivity for detecting advanced neoplasms. CRC screening experience outside the NSCP was not considered for assigning the first and the subsequent screening round. This possible misclassification is however expected to be non-differential between the first and the subsequent screening rounds.

In conclusion, the FOBT showed reasonable validity in an organized screening setting. Several factors, including screening round, use of a quantitative kit, participant's age and sex, and the anatomical locations of colorectal cancer affected FOBT sensitivity in the NCSP in Korea.
